# A Robust ORMS Framework for Taiwanese Healthcare: Taguchi’s Dynamic Method in Action

**DOI:** 10.3390/healthcare13091024

**Published:** 2025-04-29

**Authors:** Hung-Chang Liao, Ya-Huei Wang

**Affiliations:** 1 Department of Health Policy and Management, Chung Shan Medical University, Taichung 40201, Taiwan; hcliao@csmu.edu.tw; 2 Department of Medical Management, Chung Shan Medical University Hospital, Taichung 40201, Taiwan; 3 Department of Applied Foreign Languages, Chung Shan Medical University, Taichung 40201, Taiwan; 4 Department of Medical Education, Chung Shan Medical University Hospital, Taichung 40201, Taiwan

**Keywords:** operating room management system (ORMS), simulation system, optimal parameter setting, Taguchi’s dynamic method

## Abstract

The study focused on the design of an ORMS in a medical center in central Taiwan, which also functions as a teaching hospital. **Background/Objectives**: The research objectives were to design an ORMS simulation system based on the status quo of the operating room planning and scheduling in the medical center, obtain the optimal parameter setting in the ORMS, and find improvement strategies according to the sensitivity analysis based on the optimal parameter setting for total performance. **Methods**: Taguchi’s dynamic method was adopted to design the ORMS under human and material resource constraints. The scope of the study was internal medicine patients of the ORMS. A neural network was used to construct a relationship between parameters and performances. A genetic algorithm was used to obtain the optimal parameter setting for optimal performance. **Results:** This study successfully established a robust operating room management system (ORMS) to help hospital manager to plan and schedule operating rooms and take the ORMS into account to meet patient needs. Decision-makers can use the insights from the sensitivity analysis to refine their strategies effectively. The sensitivity analysis showed that the impact power (the percentage change in *d*) of the “number of circulating nurses (−0.15 to −1.25; −0.25 to −1.85)” factor was less than (<) that of the “number of holding nurses (−0.85 to −2.04; −0.91 to −2.07)” factor < that of the “number of preoperative beds (−2.57 to −4.53; −2.23 to −4.10)” factor < that of the “number of anesthetists (−3.13 to −7.50)” factor. **Conclusions**: In the optimal parameter setting obtained, the number of holding nurses was 18, the number of circulating nurses was 20, the number of anesthetists was 15, and the number of preoperative beds was 12. The optimal performance was 0.91.

## 1. Introduction

The operating room represents the largest single cost in a hospital delivering surgical care [1,2]. The high cost of medical facilities and a variety of professional medical staff, including nurses, anesthetists, and anesthesiologists, account for most of the operating room costs [3]. Hence, reducing operating room costs and increasing labor productivity is a crucial issue in hospital management. A hospital manager must continuously check how many staff and facilities are needed to care for operating room patients. However, although operating room costs are the largest single cost in a hospital, it is well known that the operating room is also the largest revenue source for hospitals [2]. Therefore, to increase operating room performance, a hospital manager should utilize strategies that enhance the hospital’s revenue while obtaining consistent patient satisfaction ratings. Adequate operating room planning and scheduling play an important role in increasing operating room performance, which the hospital manager should take into consideration. Magerlein and Martin [4] stated that in operating room management, the resolution of surgical case problems has two phases: planning and scheduling. Operating room planning focuses on making capacity decisions by assigning surgical dates to patients based on the availability of operating rooms and surgeons. Operating room scheduling mainly concerns the sequence and time of operations, determining the sequence and time allocated to each operation in each operating room on a daily basis under material and human resource constraints. Hence, hospital managers must find a way to improve operating room efficiency through planning and scheduling operating rooms to maximize the number of surgical cases and minimize the required resources and associated costs while ensuring patient satisfaction [5,6].

Operating room capacity is typically measured in planning and scheduling, including physical resources, human resources, and time availability [7]. Considering operating room scheduling and capacity planning, hospital managers must first meet the demands of surgical cases and then specify the resources needed, simultaneously considering cost-effectiveness [8]. The reason is that inadequate capacity planning decreases the quality and efficiency of the operating room. That is, if the operating room planning and scheduling are inadequate, it can be possible that the operating room does not have enough capacity to accommodate patients undergoing surgery. In that case, hospital managers must pay attention to the allocation of patient admission time or send patients to other hospitals with more capacity at that time [3,9]. Some researchers [10,11,12] have proposed operational (short-term), tactical (medium-term), and strategic (long-term) operating room planning to ensure high-quality care for patients.

Studies [9,13,14] have also examined how to expand capacity for different strategic levels. Butler et al. [15] and later Roth and van Dierdonck [16] put forward a multilevel method for planning the process for operating rooms. In operating room planning and scheduling, May et al. [7] considered the performances of each surgery to schedule or reschedule several emerging topics and update previous classification schemes. Dexter and O’Neill [9] used Data Envelopment Analysis (DEA) to compare performance in several capacity expansions, workloads, and external competitions. Dexter et al. [13] investigated the allocation of operating room time after deciding to increase the number of operating rooms at the tactical (medium-term) level. After an extensive literature review, Bai et al. [9] examined the accounting and operational factors and their interplay to bridge the gap in the capacity investment literature and empirically demonstrate their interactions in capacity investment.

In addition, two types of patients, elective and non-elective, should be considered in operating room planning and scheduling. Elective patients refer to patients for whom the operating room can be planned. Non-elective patients refer to those patients whose surgery is always unexpected and urgent. Adan and Vissers [17] constructed a mixed-integer programming model to maximize the use of different resources like operating rooms or intensive care units. Elective patients, including inpatients and outpatients, are considered in this model, but non-elective patients are not. Wullink et al. [18] examined operating room capacity. They used discrete-event simulation to assess better options: reserving an operating room or reserving a certain capacity in all elective operating rooms to meet the needs of non-elective patients who need surgery urgently to deal with emergencies and responsiveness associated with these patients. The results showed that reserved capacity can improve responsiveness to emergencies, reduce overtime, and more efficiently utilize the entire operating room. Marcon and Dexter [19] examined multiple sequencing rules and their impact on the number of patients per hour in the post-anesthesia care unit (PACU). They also evaluated the utilization of the operating room.

Lengthy waiting room time is a top patient complaint. Operating room planning and scheduling will affect waiting times. Studies have shown that a reduction in waiting time will lead to a reduction in surgical costs. Denton et al. [20] formulated a two-stage stochastic mixed-integer program (SMIP) to solve the problems of patient waiting time and operating room idling time or overtime. They proposed an effective algorithm and sensitivity analysis and applied them to decrease surgical costs. VanBerkel and Blake [21] proposed a discrete-event simulation (DES) method to decrease patient waiting time. Their results showed that throughput will also be affected by adjusting the ward bed capacity and available time of operating rooms.

The operating room utilization rate is also considered for operation room planning and scheduling. Maximizing the utilization rate means the operating room is fully used, and no unnecessary costs are incurred. However, there are no buffers when the operating room is fully planned. In an emergency where an operating room is needed, this situation will result in high costs, such as staff overtime and patient deferrals [6,22,23,24,25,26,27,28]. Strategic decisions should define the target of utilization rates. The hospital manager should decide on the target based on their experience. Van Houdenhoven et al. [29] indicated that the scheduling of the operating room will affect the use of other facilities or human resources, for instance, critical care and staff, regular ward beds, etc. Also, the utilization rate target will affect the utilization of different resources and facilities, such as bed occupancy and workload in the holding area. Applying discrete-event simulation, Marchon and Dexter [30] simulated operating room scheduling to derive optimal sequencing rules to handle the maximum number of patients in the waiting area and the PACU; for instance, the longest case should be first, or the shortest case should be first [30].

For the past studies of simulation models constructed in the operating room, Baumgart et al. [31] suggested incorporating simulation into the BPM lifecycle to enhance the efficiency of the operating room, providing evidence that simulations aid decision-making and improve performance at various stages. Persson et al. [32] employed a discrete-event simulation approach to refine operating room management, optimizing indicators such as waiting times, cancellations, and capacity utilization while managing uncertainties in patient demand and surgery length, particularly for hip joint replacement procedures. M’Hallah and Al-Roomi [33] created a simulation model to improve operating room utilization, decrease variance in completion times, and evaluate strategies such as canceling surgeries and managing queues. The model is easy to use, can be adapted to other hospitals, and supports surgeons and hospital management in making decisions. Abdullah et al. [34] used clustering to compare 34,025 cases and discrete-event simulation models to access resumption strategies, emphasizing the usage of operating rooms, clearing the backlog, and waiting times. Through a phased resumption strategy, their tool assessed the reduction in peak operating room and bed utilization while gradually extending the recovery period to return to normal operations. Schoenfelder et al. [35] designed a simulation model that enhances surgery scheduling, handles disruptions, and allocates resources more effectively, identifying the trade-offs between the time taken to wait, defer, and perform caused by scheduling changes.

In this study, to help hospital managers plan and schedule operating rooms and take the operating room management system (ORMS) into account to meet patient needs, the researchers adopted Taguchi’s dynamic method to design a robust ORMS considered under human and material resource constraints. Moreover, the objective of the ORMS was to set the duration of surgical cases and the total cost, because there exists a trade-off between time and cost. Therefore, in the design of the ORMS, time and cost were simultaneously considered to obtain the optimal ORMS, in order to minimize service time and associated costs.

## 2. The Scenario for the ORMS Simulation

To deal with the problem of operating room planning and scheduling and optimize service time and associated costs, most hospitals have started to take action to improve the space and hardware facilities and enhance their operating room staff training. Yet, the ORMS is a complex system; it is a multi-systematic problem that involves the interaction of complex factors including nurses, surgeons, and patients [36,37]. To solve the ORMS problem, in addition to relying on professional health care staff and facilities, most importantly, a resolution must be proposed to ensure correct and effective healthcare procedures and allocation of medical resources [6,38]. It requires the joint efforts and cooperation of the hospital’s internal departments to optimize the operating room treatment procedures and allocation of medical resources to make treatment more effective [39].

This study focused on the design of a robust ORMS for a medical center in central Taiwan, which also functions as a teaching hospital. Through the patients’ actual time spent during each procedure in the ORMS, the allocation of each procedure was calculated through statistics. The researchers derived the dataset used in this study from a medical center located in central Taiwan. The simulation data consist of 10,000 entries. The researchers designed the dataset based on real-world constraints provided by the hospital, ensuring its relevance to actual hospital operations. The dataset incorporates diverse factors, and the researchers also created the source referenced from a simulation system of the ORMS. Using Fei et al. [40]’s and Meskens [41]’s studies as a guide, the researchers considered the system and the real-world medical center context to ensure the data closely reflect hospital practices and environments. The researchers illustrated the assumptions for the ORMS setting as shown below:

➢ The operating rooms serve multiple functions and are specialized for different surgeries, each equipped for specific procedures.
➢ No surgeon can decide the sequence of operations for the following week.
➢ Emergencies are permitted.
➢ When a surgical procedure starts in an operating room, the procedure will not be stopped.
➢ When a surgical case starts entering the ORMS, it cannot be cancelled.
➢ Human resources are limited in the ORMS.
➢ The surgeon can operate only one case in a surgical period.
➢ The recovery bed is available in most cases but may be affected by PACU capacity limitations [42].
➢ The patients scheduled for surgery prepare for their operations on the designated surgery day.

**Figure 1:**
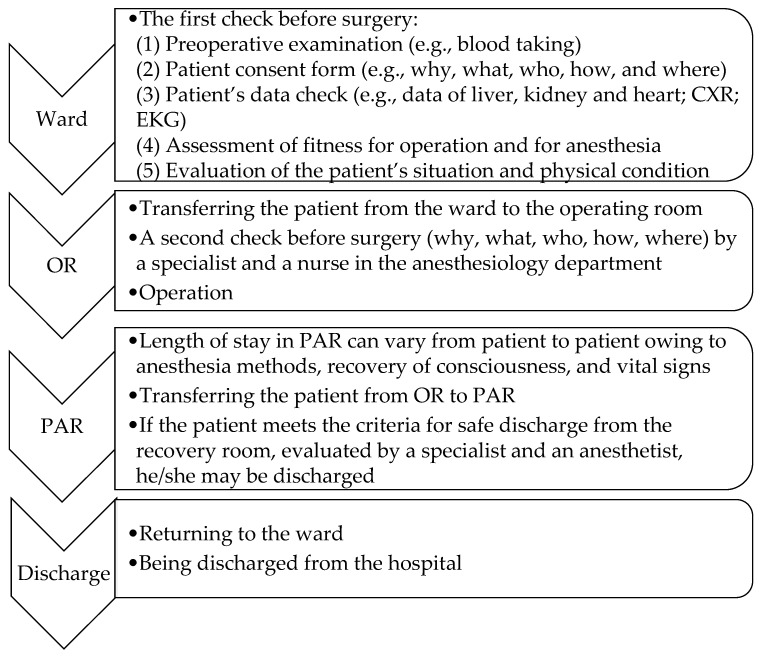
ORMS procedure.

The operating time includes the induction time, the treatment time, and the cleaning time before the patient’s leaving the operating room. Figure 1 shows the steps of the ORMS procedure. Step one is when the patient enters the ward. The tasks undertaken in the ward include the following: (1) preoperative examination, (2) patient consent form, (3) patient’s data check, (4) assessment of fitness for operation and anesthesia, and (5) evaluation of the patient’s situation and physical condition by specialists. Step two is when the patient enters the operating room (OR). The simulation design includes different configurations tailored to specific surgical specialties. The tasks undertaken in this step are a second check by a specialist and a nurse in the anesthesiology department and the operation. Step three is when the patient enters post-anesthesia recovery (PAR), and the tasks in this step are monitoring vital signs and the recovery of consciousness. Finally, the patient is returned to the ward or discharged from the hospital.

In this research, Taguchi’s dynamic method was adjusted to define the noise, signal, and control factors in order to simulate the ORMS. The noise factor was set as the patient arrival rate, the signal factor was set as the operating room utilization rate, and the control factors were set as the number of operating rooms, holding nurses, circulating nurses, anesthetists, and preoperative beds, respectively. The simulation process is shown in Figure 1. The scope of the study was internal medicine patients of the ORMS.

The signal factor was set as the operating room utilization rate. The utilization of the operating room could not be set to 100% because some operating room capacity had to be reserved for unexpected, non-elective patients, who need surgery in an emergency. Furthermore, this study considered different situations to have different utilizations of operating rooms. For example, on Friday, Saturday, and Sunday, the number of accidents is much higher than on the other days of the week. Thus, from Monday to Thursday, the operating room utilization rate was set to 85%, and from Friday to Sunday, it was set to 75%. The noise factor was assumed to be the Poisson distribution $P(x)=\frac{\lambda^{x}}{x!}e^{-\lambda }$, where λ is the surgical case arrival rate $\frac{\mathrm{patients}}{\mathrm{day}}$, and x is the number of cases on a daily basis. Here, the exponential distribution was used to transfer Poisson distribution to obtain x. The exponential distribution was set as $f(x)=\lambda e^{-\lambda x}$. F(x) is the cumulative distribution function of f(x). The research used F(x)=θ (θ was simulated for the Monte Carlo method between 0 and 1) to obtain $x_{t}$, where $x_{t}$ is the number of patients in period t. In the duration between Monday and Thursday and the duration between Friday and Sunday, the researchers set varied patient arrival rates. Thus, the noise factor was set as the patient arrival rate. The arrival rates were set to 80 patients/day and 30 patients/day, respectively. The control factors, as referred to in [43], were the number of holding nurses, the number of circulating nurses, the number of anesthetists, and the number of preoperative beds. The control factor levels were set as shown in Table 1.

The system performances included the total cost (*TC*) for each patient and the system time (*ST*) for each patient. The formulation is as follows:

(1) $$\begin{array}{l} TC=(\sum_{t=1}^{7}\sum_{i=1}^{N}({n^{o}}_{it}+{a^{o}}_{it}+{c^{o}}_{it})(X_{it}-Y_{it})x_{it}+\\ \sum_{t=1}^{7}\sum_{i=1}^{N}({n^{r}}_{it}+{a^{r}}_{it}+{c^{r}}_{it})Y_{it}x_{it}+\sum_{t=1}^{7}\sum_{i=1}^{N}b_{it}B_{it}X_{it}+\sum_{t=1}^{7}\sum_{i=1}^{N}c_{it}X_{it}/N \end{array}$$

${n^{o}}_{it}$: the overtime cost/minute of the holding nurse for patient *i* on date *t*.

${a^{o}}_{it}$: the overtime minute or cost of the anesthetist for patient *i* on date *t*.

${c^{o}}_{it}$: the overtime minute or cost of the circulating nurse for patient *i* on date *t*.

${n^{r}}_{it}$: the regular time or cost of the holding nurse for patient *i* on date *t*.

${a^{r}}_{it}$: the regular time or cost of the anesthetist for patient *i* on date *t*.

${c^{r}}_{it}$: the regular time or cost of the circulating nurse for patient *i* on date *t*.

$X_{it}$: the patient *i* on date *t*.

$Y_{it}$: the patient *i* in regular time on date *t*.

$x_{it}$: the treating time in overtime for patient *i* on date *t*.

$y_{it}$: the treating time in regular time for patient *i* on date *t*.

$b_{it}$: the preoperative bed cost/minute for patient *i* on date *t*, considering the costs of associated medical staff.

$B_{it}$: the time for preoperative bed for patient *i* on date *t*.

$c_{it}$: the treating cost for patient *i* on date *t*.

$O_{t}$: the maximal overtime on date *t*.

$R_{t}$: the regular time on date *t*.

The subject is

(2) $$\sum_{i=1}^{N}(X_{it}-Y_{it})x_{it}\leq O_{t}$$

(3) $$\sum_{i=1}^{N}Y_{it}x_{it}\leq R_{t}$$

The system time (ST) is shown in Equation (4).

(4) $$ST=({\sum_{t=1}^{7}\sum_{i}^{N}W}_{it}+\sum_{t=1}^{7}\sum_{i=1}^{N}S_{it})/N$$

$W_{it}$: the waiting time for patient *i* on date *t*.

$S_{it}$: the service time for patient *i* on date *t*.

This study assumed the waiting time for each station is in normal probability distribution, $N(\mu ,\sigma^{2})$. Hence, $W_{it}$ is $N(\sum_{j=1}^{4}\mu_{j},\sum_{j=1}^{4}\sigma_{j}^{2})$, where $\mu_{j}$ is the mean of waiting time for station *j* and $\sigma_{j}^{2}$ is the variance of waiting time for station *j*. Also, the service time for each station is in normal probability distribution. Hence, $S_{it}$ is $N(\sum_{j=1}^{4}\mu_{j},\sum_{j=1}^{4}\sigma_{j}^{2})$, in which $\mu_{j}$ is the mean of service time for *j* station and $\sigma_{j}^{2}$ is the variance of service time for *j* station. The parameter values are shown in Table 1.

## 3. Results

Based on the above presentation of Taguchi’s dynamic method, three steps were taken to attain the study objectives.

### 3.1. Step 1—To Obtain the Simulation Data Using the Orthogonal Array of Taguchi’s Dynamic Method

The orthogonal array of Taguchi’s dynamic method, $L_{9}$, was simulated to obtain the data on performance [44]. Different combinations of parameter levels were simulated: two levels used for the noise factor; two levels used for the signal factor; $L_{9}$ used for control factors. Each combination was simulated 1000 times, and the *TC* and *ST* for each simulation were obtained.

### 3.2. Step 2—To Establish a Relationship Between Parameters and Performances

Since the time and cost variables were in a normal probability distribution, to obtain the optimal parameter setting for the ORMS, this research used the neural network (NN) to set up the mathematical model [45]. The input nodes for the NN were the control factor levels, and the output node for the NN was $d=\sqrt{d_{1}*d_{2}}$, in which $d_{1}$ and $d_{2}$ were desirability values and $d_{1}$ was from normalized TC and $d_{2}$ was from normalized ST.

The normalized TC and ST were used as desirability functions [46]. This normalized value was set between 0 and 1, increasing as the desirability value of the corresponding response increased. Considering that in the responses of the ORMS system, TC and ST were STB (smaller-the-better), the desirability values $d_{1}$ and $d_{2}$ could be defined and given as in Equations (5) and (6).

(5) $$d_{1}=\{\begin{array}{c} 1,\\ \left(\frac{TC-TC_{\max}}{TC_{\min}-TC_{\max}}\right)\\ 0, \end{array},\begin{array}{c} TC\leq TC_{\min}\\ TC_{\min}\leq TC\leq TC_{\max}\\ TC\geq TC_{\max} \end{array}$$

(6) $$d_{2}=\{\begin{array}{c} 1,\\ \left(\frac{ST-ST_{\max}}{ST_{\min}-ST_{\max}}\right)\\ 0, \end{array},\begin{array}{c} ST\leq ST_{\min}\\ ST_{\min}\leq ST\leq ST_{\max}\\ ST\geq ST_{\max} \end{array}$$

For Equations (5) and (6), the bounds $TC_{\max}$ and $ST_{\max}$ represent the upper normative limit; the bounds $TC_{\min}$ and $ST_{\min}$ represent the lower normative limit. The results of the NN are shown in Table 2. The results show that 4, 3, 1 is the best ORMS NN model, and the RMSE (root mean square error) observed in training and testing is the lowest.

### 3.3. Step 3—To Use the Genetic Algorithm to Derive the Optimal Parameter Setting

This study applied a genetic algorithm (GA) to derive the optimal parameter setting in the NN [47]. Because the control factors were discrete variables, the solutions for control factors were integers between Level 1 and Level 3. The GA operational conditions were set as follows: the number of generations was set at 1000, the population size was set at 150, the crossover rate was set at 0.85, and the mutation rate was set at 0.08. The result showed that in the optimal parameter setting obtained, the number of holding nurses was 18, the number of circulating nurses was 20, the number of anesthetists was 15, and the number of preoperative beds was 12. The optimal performance was d = 0.91.

### 3.4. Step 4—To Proceed to a Sensitivity Analysis

After the GA procedure, a sensitivity analysis was performed based on the results of the most robust parameter settings. The purpose of sensitivity analysis is that decision-makers can use sensitivity analysis results to refine their strategies effectively. The scope of the sensitivity analysis for each factor is between the lower and upper levels in each factor’s optimal setting. For example, factor A’s optimal setting is 18, between Level 2, setting 15 (the lower level), and Level 3, setting 21 (the upper level); hence, the sensitivity analysis includes increasing the situation from 18 to 21 and decreasing the situation from 18 to 15. Factor C’s optimal setting is 15 in Level 1; hence, the sensitivity analysis can only increase from 15 to 18 (Level 2, setting 18; the upper level). Factor C does not have the lower level; hence, it does not have the decreasing situation. The results are shown in Table 3 and are described as follows:

1. For factor A, the number of holding nurses, when the level is increased from 18 to 21, the *d*% will decrease from −0.85 to −2.04; and when the level is decreased from 18 to 15, the *d*% will decrease from −0.91 to −2.07.
2. For factor B, the number of circulating nurses, when the level is increased from 20 to 23, the *d*% will decrease from −0.15 to −1.25; and when the level is decreased from 20 to 17, the *d*% will decrease from −0.25 to −1.85.
3. For factor C, the number of anesthetists, when the level is increased from 15 to 18, the *d*% will decrease from −3.13 to −7.50.
4. For factor D, the number of preoperative beds, when the level is increased from 12 to 15, the *d*% will decrease from −2.57 to −4.53; and when the level is decreased from 12 to 9, the *d*% will decrease from −2.23 to −4.10.

## 4. Discussion and Conclusions

This study aimed to establish a robust operating room management system (ORMS) to help hospital managers to plan and schedule surgical and operating rooms and to take the ORMS into account in meeting patients’ needs. The study focused on the design of an ORMS for a medical center in central Taiwan. This study set the optimal parameter levels to make the ORMS more robust. The ORMS scenario was first set and then simulated using Taguchi’s dynamic method to set the noise, signal, and control factors. Then, the NN and GA were used to find the optimal parameter setting, in which the number of holding nurses was 18, the number of circulating nurses was 20, the number of anesthetists was 15, and the number of preoperative beds was 12. The optimal performance was 0.91. Finally, a sensitivity analysis was used to improve the adjustable strategies in the ORMS. Based on the sensitivity analysis results, to adjust the optimal parameter settings, the impact power (the percentage change in *d*) regarding each factor upon the optimal total performance was as follows: the impact power of the “number of circulating nurses” factor was less than (<) that of the “number of holding nurses” factor < that of the “number of preoperative beds” factor < that of the “number of anesthetists” factor. This means that adjusting the parameter level of the “number of anesthetists” factor will impact the total performance more than the adjustment of any other factor. However, adjusting the “number of circulating nurses” parameter level will have less impact on the total performance.

However, this only applies to the simulation results in the ORMS of this study. Designing and evaluating medical systems using information technologies such as neural networks is the future direction, as simulations will predict system performance, serving as a basis for improving and adjusting medical systems. To discuss the feasibility of designing ORMS, Safi et al. [48] pointed out that addressing staff adoption, integration costs, and infrastructure limitations is a key factor in improving operating room efficiency and reducing costs. Many other challenges, such as policy changes, legal regulations [49], and shifts in patient demand, certainly also affect operating room management, but these factors often have higher uncertainty. They may vary depending on time, location, or circumstances [50]. Therefore, hospitals should address staff adoption, integration costs, and infrastructure limitations. For staff adoption, one significant challenge in implementing new technologies or processes in hospitals is resistance from staff. Medical professionals and support staff may be reluctant to adopt new methods due to a lack of familiarity, fear of change, or perceived increased workload. Studies have shown that staff attitudes toward new technologies can significantly predict the success or failure of healthcare interventions [48]. Hence, proper training, clear communication of the benefits, and ensuring that the new system improves workflow rather than adding burden can help encourage staff to embrace the change. Additionally, leadership engagement and involvement in the process can significantly influence adoption [51].

High integration costs may make it difficult for hospitals, particularly those with limited budgets, to adopt new systems or technologies that could improve operating room efficiency and patient satisfaction. This barrier can also delay the implementation of necessary upgrades to outdated systems [52]. Due to hospital infrastructure limitations, many hospitals, especially older ones, may not have the physical infrastructure or technological capacity to support new systems. Limited space, outdated equipment, or a lack of advanced IT systems may exist. A study by Mumtaz et al. [53] highlighted that legacy infrastructure is one of the leading barriers to upgrading hospital operations. Hospitals can prioritize incremental upgrades to their infrastructure, explore partnerships with tech companies for customized solutions, or adopt cloud-based systems that do not require heavy on-site infrastructure investments. According to a study by Mohammed [54], cloud computing and mobile technology provide scalable and cost-effective solutions for hospitals with constrained infrastructure.

The contribution of this study lies in its use of Taguchi’s dynamic method to derive the optimal parameter setting in the ORMS and provide solutions to the issues that operating rooms face in Taiwan: improving operating room efficiency and minimizing the required material and human resources and associated costs while ensuring patient satisfaction. The research design also uses NN and GA. In the “Introduction” Section, a comparison of past studies in operating room simulation revealed that most simulation models primarily use simulations to analyze data and obtain their results. Hence, the advantage of this paper is that we used Taguchi’s dynamic method, NN, and GA together.

By addressing the barriers of staff adoption, integration costs, and infrastructure limitations, hospitals in Taiwan can overcome these challenges and successfully implement improvements that will result in better resource management and improved patient care. This approach optimizes performance and emphasizes the importance of the parameters’ settings to achieve the desired outcomes. Furthermore, the concept of the optimal parameter setting and performance was emphasized in the study.

Regarding its limitations, the study was conducted at a medical center that also functions as a teaching hospital in central Taiwan. Therefore, for those hospitals with different sizes, characteristics, or organizational cultures, the findings may not be generalized to them. Moreover, the scope of the study was internal medicine patients of the ORMS; therefore, when using the ORMS for surgical and operating room planning and scheduling, researchers should also consider the patients’ background in creating an optimal ORMS that will ensure patient satisfaction while improving OR efficiency. Future studies could consider different situations in various hospitals based on the investigation of the results of parameters. For example, as referenced in [55,56,57,58,59], operating room times could be considered to follow log-normal distributions. Additionally, this ORMS could be applied in different hospital settings to evaluate its feasibility in real-world operations. The parameter settings should be adjusted according to the hospital’s size, patient needs, and resource allocation, and the system’s performance should be tested in actual operating room operations. Through gradual deployment and monitoring of key performance indicators (such as operating room efficiency, patient satisfaction, and resource utilization), the effectiveness of the ORMS implementation can be further assessed, providing practical guidance for its adaptation in various healthcare institutions.

## Figures and Tables

**Table 1:** Control factor levels in the ORMS.

Control Factor	Level 1	Level 2	Level 3
Factor A: the number of holding nurses	10	15	20
Factor B: the number of circulating nurses	15	25	35
Factor C: the number of anesthetists	15	20	25
Factor D: the number of preoperative beds	5	10	15

**Table 2:** The neural networks for the robust ORMS model.

Structures (Input Nodes, Hidden Nodes, Output Nodes)	RMSE	
	Training	Testing
4, 6, 1	0.02781	0.02644
4, 5, 1	0.02645	0.02581
4, 4, 1	0.02334	0.02217
***4***, ***3***, ***1***	***0.02211***	***0.02109***
4, 2, 1	0.02553	0.02315
4, 1, 1	0.03245	0.03126

Note: This study set the learning rate to automatically adjust between 0.01 and 0.25, set the momentum coefficient to 0.75, and set the number of iterations to 10,000. 4, 3, 1 is the best ORMS NN model.

**Table 3:** The adjusted d% in the optimal parameter levels in the most robust parameter settings.

Factor A	15	16	17	**18**	19	20	21
*d%*	−2.07	−1.15	−0.91	**0**	−0.85	−1.12	−2.04
Factor B	17	18	19	**20**	21	22	23
*d%*	−1.85	−1.12	−0.25	**0**	−0.15	−0.95	−1.25
Factor C	**15**	16	17	18			
*d%*	**0**	−3.13	−5.25	−7.50			
Factor D	9	10	11	**12**	13	14	15
*d%*	−4.10	−3.07	−2.23	**0**	−2.57	−3.33	−4.53

Factor A: the number of holding nurses. Factor B: the number of circulating nurses. Factor C: the number of anesthetists. Factor D: the number of preoperative beds. Adjusted *d*% = $\frac{d_{Level}-0.91}{0.91}$ where $d_{Level}$ is the *d* in the factor’s level. The optimal parameter setting: Factor A is 18. Factor B is 20. Factor C is 15. Factor D is 12.

## Data Availability

Data sharing is not applicable. No new data were created or analyzed in this study.

## Abbreviations

The following abbreviations are used in this manuscript:

ORMS	Operating room management system
NN	Neural network
GA	Genetic algorithm
